# Ruxolitinib does not completely abrogate the functional capabilities of TLR4/9 ligand-activated NK cells

**DOI:** 10.3389/fimmu.2022.1045316

**Published:** 2023-01-05

**Authors:** Carmen Mestre-Durán, Carla Martín-Cortázar, Blanca García-Solís, Alicia Pernas, Lidia Pertíñez, Víctor Galán, Luisa Sisinni, Laura Clares-Villa, Alfonso Navarro-Zapata, Karima Al-Akioui, Adela Escudero, Cristina Ferreras, Antonio Pérez-Martínez

**Affiliations:** ^1^ Translational Research in Pediatric Oncology, Hematopoietic Transplantation and Cell Therapy Group, Hospital La Paz Institute for Health Research (IdiPAZ), La Paz University Hospital, Madrid, Spain; ^2^ Laboratory of Immunogenetics of Human Diseases, Hospital La Paz Institute for Health Research (IdiPAZ), La Paz University Hospital, Madrid, Spain; ^3^ Department of Genetics, Institute of Medical and Molecular Genetics (INGEMM), La Paz University Hospital, Madrid, Spain; ^4^ Pediatric Hemato-Oncology Department, La Paz University Hospital, Madrid, Spain; ^5^ Faculty of Medicine, Autonomous University of Madrid, Madrid, Spain

**Keywords:** NK cells, TLR4 ligand, TLR9 ligand, ruxolitinib, JAK - STAT signaling pathway, TLR pathway

## Abstract

**Introduction:**

Natural killer (NK) cells are lymphocytes from the innate immune system part of the first defense barrier against infected and transformed cells, representing 5%-15% of peripheral blood lymphocytes. The cytotoxic capacity of NK cells is controlled by a balance between inhibitory and activating NK receptors expressed on their surface, which recognize and interact with the ligands on stressed cells. The cytokines involved in NK cell activation, proliferation, survival, and cytotoxicity are signaled mainly through the Janus kinase and signal transducer and activator of transcription proteins (JAK/STAT) pathway. NK cells are also activated in response to pathogens through Toll-like receptors (TLRs) expressed on their surface. Ruxolitinib is a specific JAK1/2 inhibitor approved for treating myelofibrosis and for steroid-refractory acute and chronic graft-versus-host disease (SR-GvHD).

**Methods:**

Purified NK cells from healthy donors were stimulated with two TOLL-like receptor ligands, LPS and CpG, in the presence of different concentrations of Ruxolitinib.

**Results:**

This study showed the effects of ruxolitinib on TLR4 and TLR9 ligand-activated NK cells from healthy donors. Ruxolitinib did not completely inhibit STAT3 phosphorylation and had a moderate effect on NK cell cytokine activation via the TLR pathway. Only the highest doses of ruxolitinib led to a decrease in the pro-inflammatory cytokines tumor necrosis factor α, interferon-γ, interleukin-6, and interleukin-1β. The cytotoxic capacity of stimulated NK cells versus K562, SEM, and MV-4-11 cell lines was reduced by increasing doses of ruxolitinib, but it was not completely abolished and we observed no major changes in degranulation capacity. Phenotypic changes were observed in activated NK cells in the presence of ruxolitinib. In a small cohort of pediatric patients treated with ruxolitinib for SR-GvHD, we observed no decrease in NK cell counts; however, further prospective studies with larger cohorts are necessary to confirm this finding.

**Discussion:**

In summary, our results showed that the functional capabilities and phenotype of NK cells activated through TLR4/9 agonists were not completely abolished by the inhibition of the JAK-STAT pathway by ruxolitinib.

## Introduction

Natural killer (NK) cells are lymphocytes from the innate immune system part of the first defense barrier against infected and transformed cells. NK cells represent 5%-15% of peripheral blood lymphocytes and are commonly defined by CD56 expression and a lack of CD3/T-cell receptor expression. Based on CD56 and CD16 expression, the best characterized NK cell subsets are CD56^bright^CD16^-^ and CD56^dim^CD16^+^ (1, 2). CD56^bright^CD16^-^ cells are less abundant in peripheral blood (<10%), are considered immature cells, produce high levels of cytokines such as interferon gamma (IFNγ), tumor necrosis factor beta (TNF-β), and granulocyte-macrophage colony-stimulating factor, and have lower cytotoxic potential than CD56^dim^CD16^+^ cells. CD56^dim^CD16^+^ NK cells are found mainly in peripheral blood and have high cytotoxic capacity (3, 4). NK cells exhibit cytotoxic capabilities similar to CD8^+^ cytotoxic T cells but do not require prior antigenic recognition by major histocompatibility complex (MHC) class I (5, 6). NK-cell cytotoxicity is mediated by several mechanisms, such as FasL/FasR interaction, TNF-related apoptosis-inducing ligand, perforin/granzyme release, and antibody-dependent cytotoxicity (7–9). The cytotoxic capacity of NK cells is controlled by a balance between inhibitory and activating NK receptors expressed on their surface that recognize and interact with the ligands on stressed cells (2, 10). NK cells include a large repertoire of activating receptors, such as natural cytotoxicity receptors (NKp30, NKp44, and NKp46); C-type lectin-like receptors (NKG2D, NKG2C, NKG2E); CD16 (FcγRIIIa); and activating killer immunoglobulin-like receptors (KIRs) such as DINAM-1, TLR3, and TLR9 (3, 5, 10). Among the inhibitory receptors, there are MHC class I-specific receptors, including inhibitory KIRs, which bind to MHC-I classic ligands and MHC-I non-classic ligands. There are also other types of inhibitory receptors, such as TIGIT, CD96, Lag-3, and TIM-3 (2, 10, 11).

The cytokines involved in NK-cell activation, proliferation, survival, and cytotoxicity are signaled mainly through the Janus kinase and signal transducer and activator of transcription proteins (JAK/STAT) pathway. The proliferation and survival of NK cells are dependent on cytokines such as interleukin (IL)-2, IL-15, and IL-18. IL-2 stimulates NK-cell differentiation and proliferation by enhancing the antitumor immune response through JAK1/3 and STAT1/3/5 (11–13). IL-12 activates STAT4 and to a lesser degree STAT1, STAT3, and STAT5. IL-15 is one of the most important cytokines in the process of regulating NK-cell maturation, development, and function (12, 14), which induces phosphorylation of STAT3 and STAT5. STAT5 is the major regulator of NK-cell development, maturation, survival, and function and is activated by cytokines such as IL-2, IL-7, and IL-15 (15, 16). IL-21 signals mainly *via* STAT1 and STAT3. Several authors have studied how a combination of cytokines can promote the formation of a population of cytokine-induced memory-like NK cells with enhanced IFNγ and functionality against tumor cells (4, 7, 11).

NK cells are also activated in response to pathogens from virus-transformed cells or tumor cells. Foreign pathogens can be recognized by pattern recognition receptors on the NK cell surface, of which, Toll-like receptors (TLRs) are the most studied and important. TLRs are expressed on all immune cells and play a role in triggering the innate immune response and prime adaptive immune responses (17–19). The TLR-mediated activation signal enhances the effector properties of NK cells (17, 20–22). In NK cells, TLRs can be found on the cell membrane (TLR1, TLR2, TLR4, TLR5, TLR6) and in intracellular compartments (TLR3, TLR7, TLR8, and TLR9). All TLRs (except for TLR3) initiate their signaling cascade through the adaptor protein Myd88 (17, 20). Various ligands bind to the TLRs, with differing responses. Bacterial lipopolysaccharide (LPS) and oligodeoxyribonucleotides containing non-methylated CpG motifs (CpG ODNs) are TLR agonists that bind to TLR4 and TLR9, respectively, activating signaling pathways that upregulate the expression of cytokines with the capacity to increase NK-cell cytotoxicity (23, 24). TLR activation has been suggested as an alternative route for reestablishing the antitumor activity of NK cells (21).

Ruxolitinib (Novartis, INCB018424) is a specific inhibitor of the JAK/STAT pathway that has been approved for treating myelofibrosis, a rare myeloproliferative neoplasm (25), and as a treatment for steroid-refractory acute and chronic graft-versus-host disease (GvHD) in adult and pediatric patients aged 12 years and older (26, 27). GvHD remains an obstacle to improving the success rate of allogeneic hematopoietic stem cell transplantation (allo-HSCT). GvHD is a complex process primarily involving donor alloreactive T cells; however, emerging data have shown a more complicated scenario with TLR activation in antigen-presenting cells (APCs) involved in GvHD disease. Chemotherapy or a conditioning regimen to remove tumor cells and allow engraftment also causes intestinal tissue damage, provoking the release of the bacterial components from gastrointestinal epithelial cells. These signals activate APCs, producing pro-inflammatory cytokine release and activation of donor alloreactive T cells, triggering or aggravating GvHD (28–30). TLR4 and TLR 9 have been the subject of study for many years for their role in GvHD, with TLR4 activation by LPS considered a driver of GvHD in murine models (28). NK cells are the first lymphocyte subset to reconstitute after allo-HSCT, providing a barrier against viral and bacterial infections and preventing relapse during T-cell deficiency (31, 32). One of the main benefits offered by allo-HSCT is the graft-versus-leukemia (GvL) effect. The decrease in the occurrence of GvHD can lead to graft failure and a reduction of the GvL effect. Data show that alloreactive NK cells from donors greatly promote GvL (11). Although ruxolitinib interacts with the proliferation and cytotoxicity of cytokine-activated NK cells (11, 25), little is known about the role of ruxolitinib in NK-cell activation *via* TLR ligands. In this study, we analyzed the phenotype and functional capacities of highly purified NK cells from healthy donors that have been activated by TLR4 and TLR9 ligands and the effect of ruxolitinib on the NK cells’ phenotype, cytokine release, and killing capacity.

## Materials and methods

### Natural killer-cell purification and cell culture

We purified NK cells from the buffy coats of healthy donors using RosetteSep Human NK Cell Enrichment Cocktail (STEMCELL Technologies; 15065) and Ficoll centrifugation (GE Healthcare), following the manufacturer’s instructions. After enrichment we obtained a mean of 90% of CD56^+^ CD3^-^ (CD45^+^) cells (**Figures S1**, **S2**) of the final product by flow cytometry (Navios Flow Cytometer, Beckman Coulter). We cultured NK cells in TexMacs medium (Miltenyi Biotec) supplemented with 10% human AB serum (Sigma-Aldrich) and 1% penicillin/streptavidin (Sigma-Aldrich), and maintained them at 37°C in a humidified incubator with 5% CO_2_.

The K562 cell line was kindly provided by Dr. Julian Pardo (Aragón Health Research Institute) and was cultured in RPMI medium (Lonza) supplemented with 10% fetal bovine serum (ThermoFisher), 1% penicillin/streptavidin, 1% GlutaMax, 1% non-essential aminoacids and 1% sodium pyruvate (Sigma-Aldrich) and maintained at 37°C in a humidified incubator with 5% CO_2_. K562 is derived from a chronic myelogenous leukemia patient and is highly sensitive to NK cells killing due to the lack of HLA and high expression of the NKG2DL (33). SEM and MV-4-11 cells lines were from the Leibniz-Institut DSMZ and were cultured in Iscove’s Modified Dulbecco’s Medium (IMDM, Cytiva) supplemented with 10% fetal bovine serum (ThermoFisher), 1% penicillin/streptavidin, 1% GlutaMax, 1% non-essential aminoacids and 1% sodium pyruvate (Sigma-Aldrich) and maintained at 37°C in a humidified incubator with 5% CO2. MLL-rearranged leukemia cell lines (SEM and MV-4-11) are usually resistant to NK cell lysis since expressing some inhibitory KIR ligands and low levels of the activating receptor NKG2D (34).

### Expression of pro-inflammatory cytokines by quantitative reverse transcription polymerase chain reaction

We plated NK cells at 2 × 10^6^ cells/mL in 6-well plates and cultured them at 37°C in complete media: TexMacs (Miltenyi Biotec) supplemented with 10% human AB serum (Sigma-Aldrich) and 1% penicillin/streptavidin (Sigma-Aldrich), and incubated them overnight with 100 ng/mL LPS (Sigma-Aldrich) and 10 µg/mL aCpG (ODN 2216, Miltenyi Biotec). When appropriate, increasing concentrations of ruxolitinib (0.1 µM, 1 µM, 10 µM) (Novartis) were added. The concentration of TLR4/9 ligands used was based on a publication by Pérez-Martinez et al. (22). After the incubation, we isolated RNA from the NK cells using the RNeasy Mini Kit (Qiagen), and cDNA was obtained with the SuperScript IV First-Strand Synthesis System (Invitrogen). We amplified the cDNA using specific primers for human TNFα (Hs00174128_m1), IL-6 (Hs00174131_m1), IL-1β (Hs01555410_m1), and IFNγ (Hs00989291_m1) with their respective probes (Applied Biosystems), GUS (Fw: 5′-GAAAATATGTGGTTGGAGAGCTCATT−3′, Rv: 5′-CCGAGTGAAGATCCCCTTTTTA−3′; Probe: 5′-[6FAM] CCAGCACTCTCGTCGGTGACTGTTCA[TAMRA]−3′; all from Sigma), and TaqMan Universal PCR Master Mix (ThermoFisher). We performed real-time quantitative polymerase chain reaction with a LightCycler 480 (Roche) and analyzed the relative expression employing the 2−ΔCT method, in which ΔCT = (Ct gene of interest- Ct internal control).

### Production of pro-inflammatory cytokines by enzyme-linked immunosorbent assay

We plated NK cells at 2 × 10^6^ cells/mL in 6-well plates and incubated them overnight with 100 ng/mL LPS and 10 µg/mL aCpG in the presence or absence of increasing concentrations of ruxolitinib (0.1 µM, 1 µM, 10 µM) at 37°C in complete media. We measured the cytokines TNFα, IL-6, IL-1β, and IFNγ in the supernatant using the ELISA MAX Deluxe Set Human TNFα, IL-6, IL-1β, and IFNγ, respectively, according to the manufacturer’s instructions (Biolegend). We analyzed the cytokines with a BioTek Synergy HTX Microplate Reader (Biotek).

### Cell-mediated cytotoxicity assays

We evaluated the cytotoxic capacity of TLR4/9 ligand-activated NK cells using DELFIA Cell Cytotoxicity Assays (PerkinElmer). We incubated NK cells (2 × 10^6^ cells/mL) overnight with 100 ng/mL LPS and 10 µg/mL aCpG in the presence or absence of increasing concentrations of ruxolitinib (0.1 µM, 1 µM, 10 µM) at 37°C in complete media. After incubation, we washed the NK cells, then stimulated them with BATDA-labeled tumor cell lines K562, SEM, and MV-4-11 (10^6^ cells/mL), as indicated by the manufacturer. We co-cultured the BATDA-labeled cell lines (5000 cells/well) with the NK cells at the indicated effector:target (E:T) ratios (20:1, 10:1, 5:1) at 37°C for 4 hours in complete media. We added various controls to measure the BATDA-associated background (harvested tumor cell supernatant after labeling), spontaneous tumor cell lysis (without NK cells), and maximum cellular lysis (lysis of tumor cells with 10% Triton X-100), performing the experiments in triplicate. We employed an Infinite 2000 Reader (Tecan) to measure the Europium signal and calculated the percentage of specific killing as 100 X (experimental lysis-spontaneous lysis/maximum lysis-spontaneous lysis).

### Degranulation assay

We incubated purified NK cells (2 × 10^6^ cells/mL) overnight with 100 ng/mL LPS and 10 µg/mL aCpG in the presence or absence of increasing concentrations of ruxolitinib (0.1 µM, 1 µM, 10 µM) at 37°C in complete media. After incubation, we stimulated the NK cells with the tumor cell lines K562, SEM, and MV-4-11 at 1:1 and 1:2 E:T ratios at 37°C for 4 hours in complete media with the specific antibody anti-CD107a (APC, Miltenyi Biotec) and Golgi Stop (BD Biosciences). We then stained the NK cells with 7-aminoactinomycin D (7-AAD) and specific antibodies against the surface receptors CD45 (BV510, Biolegend), CD3 (PCy7, Biolegend), CD16 (APC-Cy7, BD Biosciences), and CD56 (AlexaFluor770, BD Biosciences). We performed a flow cytometry analysis on a Navios Flow Cytometer and employed FlowJo v10.0.7 software (BD Biosciences) for the data analysis.

### Natural killer-cell proliferation assay

Purified NK cells from buffy coats were labeled for 10 min at 37°C with 5 µM carboxyfluorescein succinimidyl ester (CFSE, BD Bioscience) in the dark. We washed the NK cells twice with phosphate-buffered saline 1X, then incubated them overnight with 100 ng/mL LPS and 10 µg/mL aCpG in the presence or absence of increasing concentrations of ruxolitinib (0.1 µM, 1 µM, 10 µM). To stimulate proliferation, we added 100 IU/ml IL-2 (Miltenyi Biotec). We plated NK cells at 1 × 10^6^ cells/mL in 12-well plates and cultured them at 37°C in complete media. After 6 days, we collected and labeled the cells with 7-AAD, CD3 (APC, BD Pharmingen), CD56 (AlexaFluor770, BD Biosciences), and CD45 (BV421, Biolegend), and acquired them on a Navios Flow Cytometer (Beckman Coulter). We calculated the division index using the total number of divisions divided by the number of cells at the beginning of the culture, employing FlowJo v10.0.7 software (BD Biosciences).

### Immunophenotyping of natural killer cell receptors

We investigated the immunophenotyping of functionally relevant receptors within NK subpopulations by specific staining and posterior flow cytometry. We incubated purified NK cells (2 × 10^6^ cells/mL) overnight with 100 ng/mL LPS and 10 µg/mL aCpG in the presence or absence of increasing concentrations of ruxolitinib (0.1 µM, 1 µM, 10 µM) at 37°C in complete media. After incubation, we performed immunophenotyping of functionally relevant receptors within the NK subpopulations with TIGIT PE-Vio770, CD69 BV421, TACTILE BV421 (Biolegend), CD56 AlexaFluor770, CD16 APC-Cy7, CD25 FITC, DNAM-1 PE, CXCR4 APC, NKG2D BV421, KIR3DL1 PE-Vio770, KIR2DL2/L3/S2 FITC, KIR3DL2 PE (BD Biosciences), NKp46 FITC, NKp44 PE, NKp30 APC, CD57 FITC, KIR2DS4 PE, NKG2A PE-Vio615, PD-1 FITC, LAG-3 PE, TIM-3 PE-Vio770, KIR2DL4 APC, KIR2DL1 BV421, CD3 Viogreen (Miltenyi Biotec), and NKG2C APC (R&D Systems). We acquired samples in a Navios flow cytometer (Beckman Coulter) and employed FlowJo v10.0.7 software (BD Biosciences) for the data analysis.

### Western blotting

We incubated purified NK cells (2 × 10^6^) with 100 ng/mL LPS and 10 µg/mL aCpG in the presence or absence of increasing concentrations of ruxolitinib (0.1 µM, 1 µM, 10 µM). In addition, we used 100 IU/ml IL-2 and 100 µg/mL IFNγ (Sigma Aldrich) as positive controls for a canonical activation of the JAK-STAT pathway. We harvested the NK cells and suspended them in total lysis buffer (0.125 M Tris-HCl pH 6.8, 4% sodium dodecyl sulfate [SDS], and 20% glycerol). We boiled the cell lysates for 15 min and determined the protein concentration by the Lowry method. After quantification, we added β-mercaptoethanol (1:100 v/v) and bromophenol blue powder. Protein samples were resolved in 10% SDS-PAGE and transferred to nitrocellulose membranes. We blocked the membranes in 5% bovine serum albumin (Sigma-Aldrich) and Tris-buffered saline with 0.1% Tween, then probed the membranes with antibodies against phospho-STAT5 (Try694) and phosphor-STAT3 (Try705), STAT5, STAT3, and MyD88 (Cell Signaling Technologies), followed by appropriate peroxidase-conjugated secondary antibodies (Cell Signaling Technologies). To measure the chemiluminescent signal, we employed the HRP Chemiluminescent Substrate Reagent Kit (InvitroGen) and a Uvitec Imaging system (Uvitec).

### Patients

We retrospectively studied 12 pediatric patients from La Paz University Hospital (Madrid) with hematological disease who underwent allo-HSCT between March 2017 and July 2022 and who received ruxolitinib under compassionate use for treating steroid-refractory GvHD. Patients were divided into 2 cohorts according to the mean time of treatment with ruxolitinib. The first cohort received ruxolitinib for less than 6 months (comprising N=10 out of 12 patients enrolled), whereas the second cohort received treatment for more than 6 months (comprising N=8 out of 12 patients enrolled). All patients or legal guardians signed an institutionally approved informed consent document including the use of information from the medical records for research purposes. Approval for the study was obtained from the Ethics Committee of La Paz University Hospital.

### Statistical analysis

The quantitative data are shown as mean ± standard error of the mean (SEM). We employed a one-way non-parametric analysis of variance (Friedman test) for comparing the paired samples, using GraphPad Prism (version 8.0.0 for Windows, GraphPad Software, San Diego, CA, USA). The patients’ data are shown as median and interquartile range (IQR). Differences were considered statistically significant when the p-value was <0.05.

## Results

### Effect of ruxolitinib on STAT3 phosphorylation in TLR4/9 ligand-activated natural killer cells

**Figure 1:**
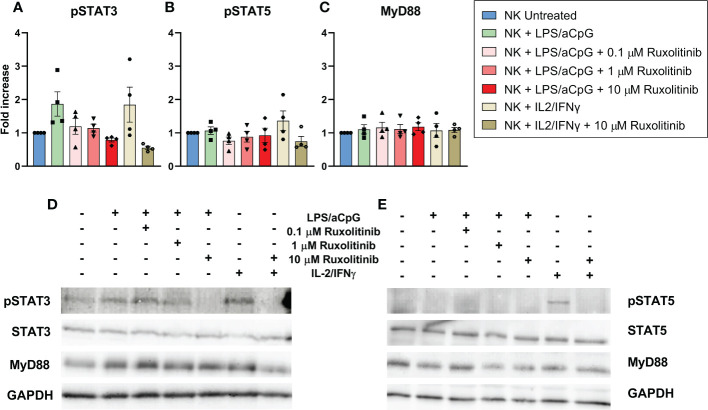
Western blot analysis and quantification of STAT3 phosphorylation **(A)**, STAT5 phosphorylation **(B)** and Myd88 expression **(C)** in NK cells from healthy donors. Phosphorylation and MyD88 expression were analyzed in NK cells activated with 100 ng/mL LPS and 10 µg/ml aCpG in the absence or presence of increasing concentrations of ruxolitinib for an overnight incubation. IL-2/IFN-γ was used as a positive control. **(A–C)** Show the quantification of pSTAT3, pSTAT5 and MyD88 respectively of four different experiments (N=4 from 4 different healthy donors). The data are shown as mean +/- SEM for independent experiments. **(D, E)** shows an example of two western blot membranes. Fold increase was defined as the ratio of TLR4/9 ligand-activated NK cells with or without ruxolitinib to untreated NK cells. All conditions were previously normalized to GADPH.

Although not significant, we observed that TLR4/9 ligand-activated NK cells led to an increase in pSTAT3. We detected that only the highest concentration of ruxolitinib showed a decrease in pSTAT3 **(** **Figures 1A, D**). The TLR4/9 ligand-activated NK cells showed no STAT5 phosphorylation, indicating that independent JAK/STAT5 signaling could be involved (**Figures 1B, E**). Treatment with IL-2-IFNγ showed a pSTAT3 and pSTAT5 activation that was abrogated at the highest dose of ruxolitinib (**Figures 1A, B, D** **, E**). MyD88 is an adapter protein of the TLR pathway employed by all TLRs to activate nuclear factor kappa B and mitogen-activated protein kinases for inflammatory cytokine gene induction (17). We observed no changes in MyD88 expression in the presence of ruxolitinib; therefore, the changes observed in the presence of ruxolitinib in pSTAT3 were independent of the TLR pathway (**Figure 1C**). Our data showed that low concentrations of ruxolitinib did not completely inhibit STAT3 phosphorylation in TLR4/9 ligand-activated NK cells.

### Effect of ruxolitinib on the expression of pro-inflammatory cytokines in TLR4/9 ligand-activated natural killer cells

**Figure 2:**
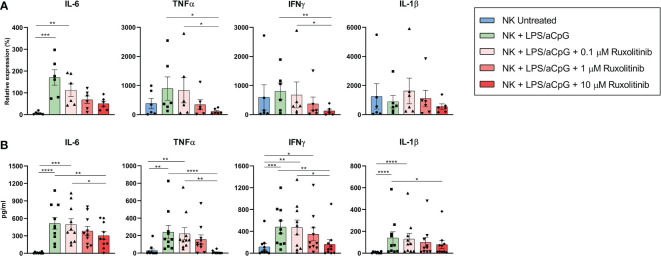
Expression of pro-inflammatory cytokines IL-6, TNFα, IFNγ, and IL-1β on TLR4/9 ligand-activated NK cells in the absence or presence of increasing concentrations of ruxolitinib. **(A)** mRNA expression levels of pro-inflammatory cytokines by RT-PCR in NK cells activated for 16 hours with 100 ng/mL LPS and 10 µg/ml aCpG in the absence or presence of increasing concentrations of ruxolitinib (0.1, 1, and 10 μM). N=6 from 6 different healthy donors. The data represent the relative expression of each cytokine after normalization with GUS levels. **(B)** Expression of pro-inflammatory cytokines by ELISA in NK cells activated for 16 hours with 100 ng/mL LPS and 10 µg/ml aCpG in the absence or presence of increasing concentrations of ruxolitinib (0.1, 1, and 10 μM). N=10 from 10 different healthy donors. The data are shown as mean +/- SEM for independent experiments. *, p < 0.05; **, p < 0.01; ***, p < 0.001, ****, p < 0.0001.

We examined the impact of ruxolitinib therapy on NK-cell pro-inflammatory cytokine production by reverse transcription-polymerase chain reaction (**Figure 2A**). TLR4/9 ligand-activated NK cells significantly increased the expression of the pro-inflammatory cytokine IL-6. We also observed a tendency toward increased levels of the pro-inflammatory cytokines TNFα and IFNγ. No differences were observed for IL-1β. We investigated whether the presence of ruxolitinib could inhibit NK cytokine production by TLR4/9 ligands. We observed that increasing concentrations of ruxolitinib reduced the mRNA expression of the pro-inflammatory cytokines IL-6, TNFα, and IFNγ in a dose-dependent manner, with significant values for TNFα and IFNγ only at 10 µM of ruxolitinib. Interestingly, only at this concentration there was a decrease in pro-inflammatory cytokine expression similar to that of untreated cells in TNFα, IFNγ, and IL-1β (**Figure 2A**). We then analyzed the expression of the pro-inflammatory cytokines in the supernatant of the NK cell cultures by enzyme-linked immunosorbent assay (**Figure 2B**). We observed a significant increase in the release of pro-inflammatory cytokines by TLR ligands for all the cytokines studied. Although these values were diminished in a dose-dependent manner in the presence of ruxolitinib, this reduction was significant only for the highest concentration of ruxolitinib. The reduction in expression with ruxolitinib was always above the value for untreated NK cells; values similar to untreated NK cells were observed for TNFα only at 10 µM of ruxolitinib (**Figure 2B**).

These data show that the increased production of pro-inflammatory cytokines through TLR4/9 ligand-activated NK cells was reduced but not eliminated in the presence of ruxolitinib.

### Impact of ruxolitinib on the cytotoxic capacity of TLR4/9 ligand-stimulated natural killer cells

**Figure 3:**
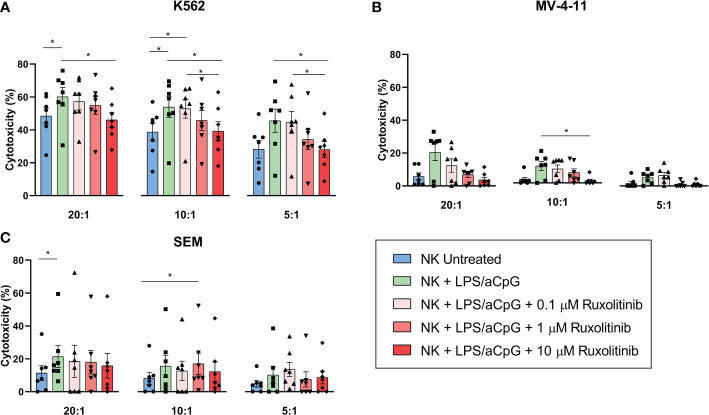
Cytotoxic capacity of TLR4/9 ligand-activated NK cells in the absence or presence of increasing concentrations of ruxolitinib (0.1, 1, and 10 μM). Cytotoxic capacity of NK cells was measured against **(A)** K562, **(B)** MV-4-11, and **(C)** SEM tumor cell lines at different E:T ratios (20:1, 10:1, and 5:1) using delfia cell cytotoxicity assay after 4 hours incubation at 37°C. N=7 from 7 different healthy donors. The data are shown as mean +/- SEM for independent experiments. *, p < 0.05.

We then sought to analyze whether ruxolitinib had an effect on the cytotoxic activity of TLR4/9 ligand-activated NK cells (**Figure 3**). We tested the cytotoxic activity of NK cells against 3 leukemia target cell lines: K562 derived from a patient with chronic myelogenous leukemia and highly sensitive to NK-cell killing due to a lack of HLA and high NKG2DL expression; and the mixed-lineage leukemia (MLL)-rearranged cell lines MV-4-11 and SEM. MLL-rearranged cell lines are typically resistant to NK-cell lysis, given that they express certain inhibitory KIR ligands and low levels of the activating receptor NKG2D. As expected, the NK cells showed different killing activity against the 3 cell lines tested (**Figures 3A** **–C**). The cytotoxic capacity of TLR4/9 ligand-activated NK cells significantly increased for K562 at 20:1 and 10:1 ratios compared with the untreated NK cells. For the SEM cell line, this activation was only significant at a 20:1 ratio. Ruxolitinib slightly reduced NK-cell cytotoxicity in a dose-dependent manner, with values similar to the untreated NK cells only at 10 µM for the K562 cell line (**Figure 3A**). The same tendency was observed for the MV-4-11 cell line, with ruxolitinib only affecting the lysis capacity at high concentrations, with significant values at a 10:1 ratio for 10 µM (**Figure 3B**). Ruxolitinib had barely any effect on the SEM cell line; a slight dose-dependent decrease in the cytotoxic capacity of NK cells was observed only at a 20:1 ratio (**Figure 3C**).

Collectively, these results showed that the cytotoxic capacity of TLR4/9 ligand-activated NK cells in 3 different leukemic cell lines was not severely affected by JAK1/2 pathway inhibition with ruxolitinib.

### Impact of ruxolitinib on the TLR4/9 ligand-stimulated natural killer cell degranulation capacity

**Figure 4:**
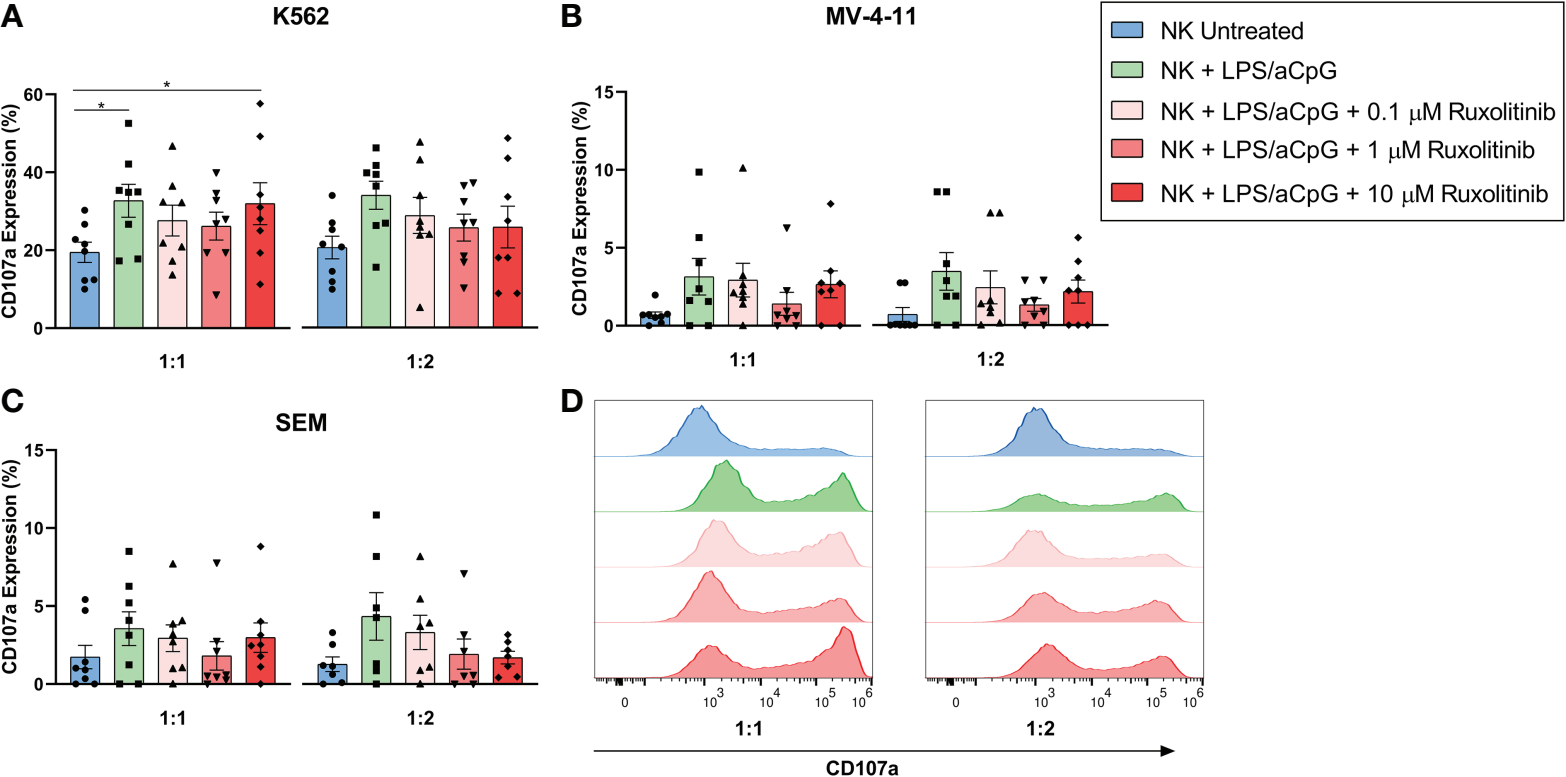
Degranulatory capacity of TLR4/9 ligand-activated NK cells in the absence or presence of increasing concentrations of ruxolitinib (0.1, 1, and 10 μM). Degranulation capacity was measured by co-culturing the cells with **(A)** K562, **(B)** SEM and **(C)** MV-4-11 tumor cell lines at 1:1 and 1:2 E:T ratios. CD107a expression was determined at the indicated E:T ratios. **(D)** Representative example of CD107a expression of the different tested conditions against K562 by flow cytometry is shown. N=8 from 8 different healthy donors. The data are shown as mean +/- SEM for independent experiments. *, p < 0.05.

To further analyze the effector functions of TLR4/9 ligand-activated NK cells, we evaluated the effect of increasing doses of ruxolitinib on the degranulation capacity of NK cells by co-culturing these cells with K562, SEM, and MV-4-11 cell lines (**Figure 4**). We found that TLR4/9 ligand-activated NK cells had the highest degranulation capacity in the 3 cell lines tested at ratios 1:1 and 1:2 and that this increase was only significant for the K562 cell line at a 1:1 ratio. The presence of increasing doses of ruxolitinib did not significantly reduce the CD107a expression. Also, a 10-µM dose of ruxolitinib did not have a major effect on degranulation capacity. These data showed that, as we observed for the cytotoxicity assay, ruxolitinib did not have much of an effect on the degranulation capacity of TLR4/9 ligand-activated NK cells.

### Effect of ruxolitinib on the proliferation of natural killer cells activated with TLR4/9 ligands

**Figure 5:**
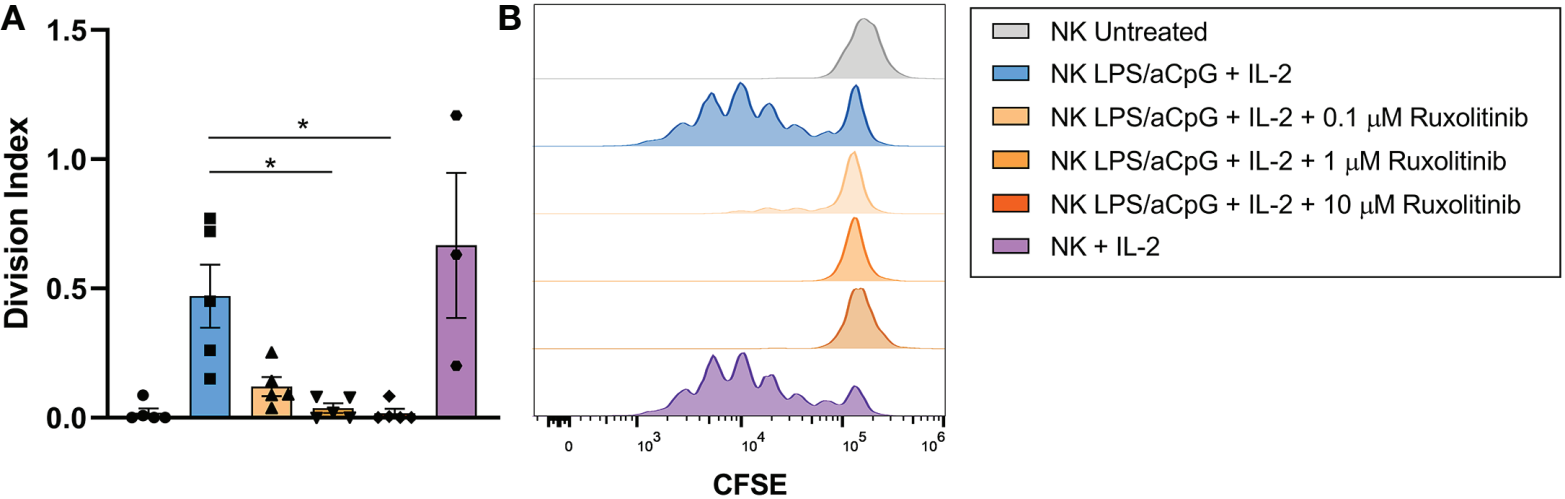
Proliferation capacity of TLR4/9 ligand-activated NK cells in the absence or presence of increasing concentrations of ruxolitinib (0.1, 1, and 10 μM). NK cells were labeled with CFSE and treated overnight with 100 ng/mL LPS and 10 µg/ml aCpG in the absence or presence of ruxolitinib (0.1, 1, 10 µM). Cells were incubated with IL-2 for 6 days. **(A)** The division index was determined using the total number of divisions divided by the number of cells at the beginning of the culture. **(B)** Representative example cell division (as assessed by reduction in CFSE staining) was determined by flow cytometry. N=5 from 5 different healthy donors. The data are shown as mean +/- SEM for independent experiments. Statistical analysis was performed using the Friedman test. *, p < 0.05.

We then studied the proliferative capacity of TLR4/9 ligand-activated NK cells and/or IL-2 after 6 days of CFSE staining in the presence or absence of increasing doses of ruxolitinib (**Figure 5** **).** No cell proliferation was observed in NK cells untreated or activated by TLR4/9 ligands (data not shown). NK-cell activation through the TLR pathway and IL-2 resulted in an increased proliferation capacity of NK cells compared with untreated NK cells. Ruxolitinib treatment reduced the proliferative activity of activated NK cells in a dose-dependent manner, as shown by a decrease in the cell division index (**Figure 5A**). The proliferative capacity of NK cells in the presence of the TLR ligand and IL-2 was lower than that obtained for cytokine-activated NK cells only (**Figure 5B**). The proliferation capacity of activated NK cells with TLR4/9 ligands and IL-2 was completely abrogated at the highest concentrations of ruxolitinib (1 and 10 µM).

### Effect of ruxolitinib on the phenotype of TLR4/9 ligand-stimulated natural killer cells

**Figure 6:**
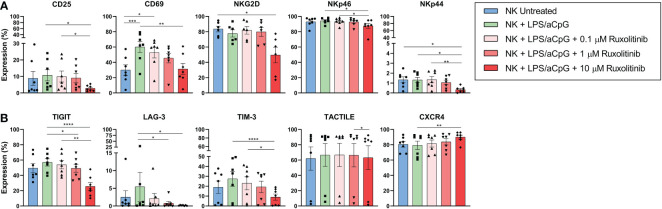
Receptors’ expression in TLR4/9 ligand-activated NK cells in absence or presence of increasing concentrations of ruxolitinib (0.1, 1, and 10 μM). NK cells were activated for 16 hours with 100 ng/mL LPS and 10 µg/ml aCpG in the absence or presence of the indicated concentrations of ruxolitinib. We analyzed changes in the activating receptors **(A)** CD25, CD69, NKG2D, NKp46, NKp44, and the inhibitory receptors **(B)** TIGIT, LAG-3, TIM-3, and TACTILE and chemokine receptor CXCR4 by flow cytometry. N=7 from 7 different healthy donors. The data are shown as mean +/- SEM for independent experiments. *, p < 0.05; **, p < 0.01; ***, p < 0.001; ****, p < 0.0001.

Given that we confirmed that ruxolitinib affected proliferation but not functional capabilities, we decided to analyze the changes in NK cell phenotype when stimulated with TLR4/9 ligands. Of the activating receptors studied, we observed an increase only in the expression of the CD69 receptor. Although the expression decreased dose-dependently in the presence of ruxolitinib, values similar to the untreated NK cells were only reached at 10 µM. CD25 expression barely increased after TLR4/9 ligand-stimulated NK cells, and only at concentrations of 10 µM did ruxolitinib decrease this receptor’s expression. The expression of NKG2D, NKp46, and NKp44 did not increase in TLR4/9 ligand-activated NK cells, and only the highest dose of ruxolitinib had an effect on the expression of NKG2D and NKp44 (**Figure 6A** and **Figure S3**). For the inhibitory receptors, we observed a tendency for expression of the immune checkpoints TIGIT, LAG-3, and TIM-3 to be increased in TLR4/9 ligand-activated NK cells. Only the highest concentration of ruxolitinib could significantly decrease the expression of TIGIT, LAG-3, and TIM-3. CXCR4 expression did not increase by TLR activation, and, contrary to the expression of the other receptors, the expression of CXCR4 increased at 10 µM of ruxolitinib (**Figure 6B** and **Figure S4**). Regarding the other receptors analyzed (DNAM-1, CD57, NKp30, NKG2C, NKG2A, PD-1, KIR2DS4, KIR2DL4, KIR3DL1, Tactile, and KIR3DL2), we observed no expression changes (data not shown).

**Figure 7:**
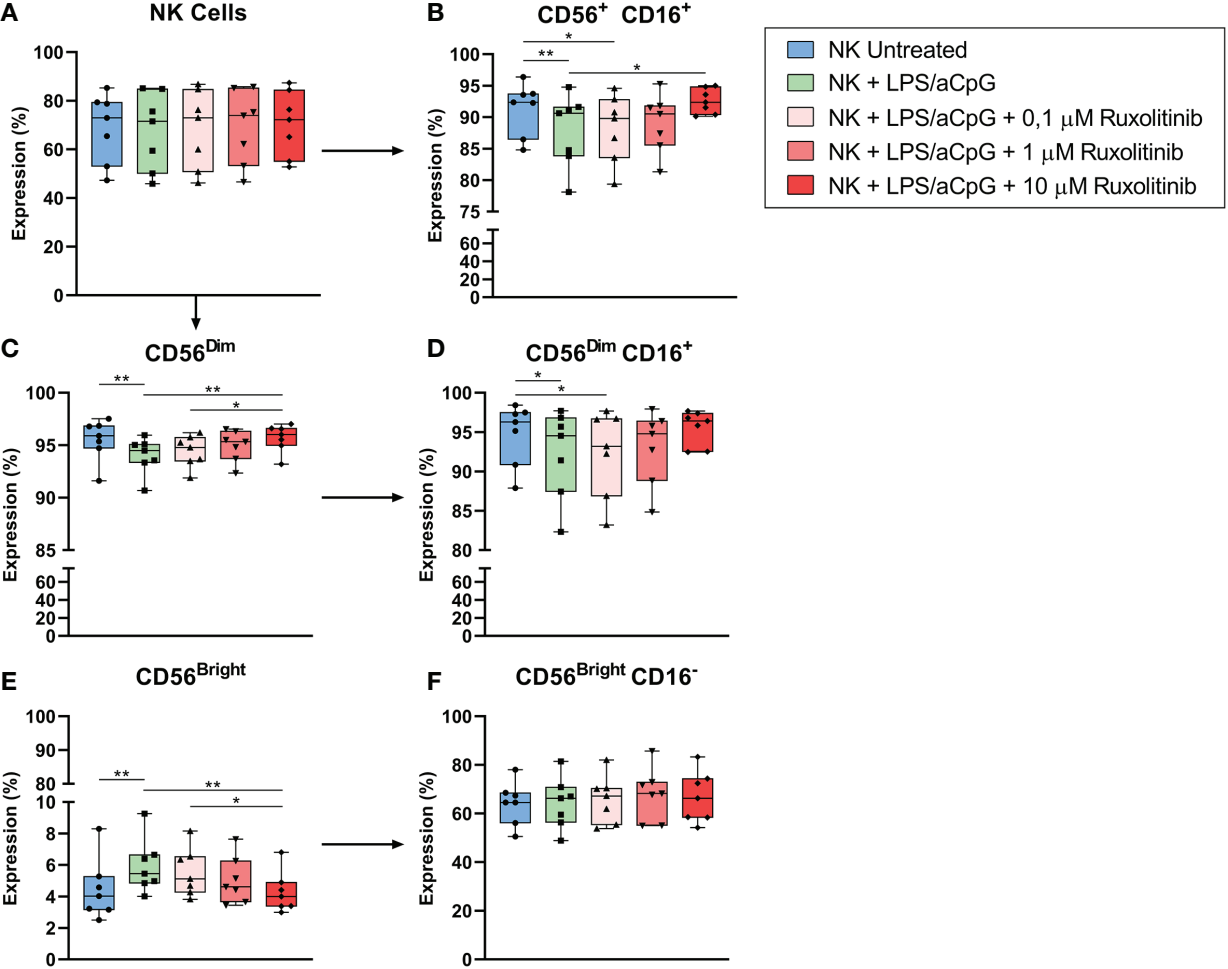
Expression changes of total NK cells and NK subsets in TLR4/9 ligand-activated NK cells in the absence or presence of increasing concentrations of ruxolitinib (0.1, 1, and 10 μM). **(A)** Percentage of expression of CD56^+^ cells, **(B)** CD56^+^ CD16^+^ subset, **(C)** CD56^Dim^ subpopulations, **(D)** CD56^Dim^ CD16^+^ subset, **(E)** CD56^Bright^ subpopulations, **(F)** CD56^Bright^ CD16^-^ subset by flow cytometry. N=7 from 7 different healthy donors. The data are shown as mean +/- SEM for independent experiments. *, p < 0.05; **, p < 0.01.

We then analyzed the effect of ruxolitinib on the percentage of NK cells. The gating strategy is shown in **Figure S5**. Neither TLR4/9 ligand activation nor ruxolitinib had any effect on the total number of NK cells (**Figure 7A**). CD16^+^ expression decreased after TLR-ligand NK-cell activation, and the highest doses of ruxolitinib increased the percentage of expression, reaching values similar to those of the untreated NK cells (**Figure 7B**). We analyzed the CD56^bright^ and CD56^dim^ subpopulations in total NK cells, observing that the TLR4/9 ligand-activated NK cells increased the CD56^bright^ subpopulation and decreased the CD56^dim^ subpopulation in the presence of ruxolitinib in a dose-dependent manner, changing their expression pattern, similarly to untreated cells (**Figures 7C, E**). Furthermore, we examined the best characterized NK cell subsets, CD56^bright^CD16^-^ and CD56^dim^CD16^+^, in TLR4/9 ligand-activated NK cells in the presence or absence of ruxolitinib. We observed a decrease of TLR4/9 ligand-activated CD56^dim^CD16^+^ cells compared with untreated cells (**Figure 7D**). Different doses of ruxolitinib did not change the expression of the CD56^bright^CD16^-^ and CD56^dim^CD16^+^ subsets (**Figures 7D, F**).

### Immune reconstitution of lymphocyte subpopulations in pediatric patients with GvHD undergoing ruxolitinib treatment

We retrospectively studied the immune reconstitution of a pediatric cohort of 12 patients who received ruxolitinib for compassionate use after HSCT. We analyzed changes in NK cell numbers, dividing the patients into 2 groups based on when they underwent ruxolitinib treatment until the sample collection (**Table 1**). We observed a tendency for higher NK cell numbers in the patients who received ruxolitinib for less than 6 months compared with pre-treatment; however, the results were not statistically significant (0.29 NK cells/µL vs. 0.09 NK cells/µL p=0.26). The patients who received ruxolitinib for more than 6 months showed NK cell values similar to pre-treatment (0.09 NK cells/µL vs. 0.095 NK cells/µL) (**Table 1**). We analyzed the fold change in NK cell levels from the beginning of HSCT at various post-transplantation time points for each patient and found no association between ruxolitinib administrations at early stages of immune reconstitution with changes in the fold change of NK cells in patients after HSCT (**Figure S6**).

**Table 1:** Immune reconstitution median (IQR) before and after treatment with ruxolitinib during 6 and 12 months with treatment.

Variable	Pre-Treatment	≤6 meses	6-12 meses
Patient Number	n=12	n= lO	n=8
Lymphocytes/µL	1.23 (0.67-1.94)	1.02 (0.69-1.58)	1.09 (0.85-1.17)
T Lymphocytes/µL	1.08 (0-0.04)	0.62 (0.34-1.08)	0.72 (0.62-0.91)
CD4+ Helper T-cells/µL	0.33 (0.27-0.69)	0.25 (0.15-0.43)	0.35 (0.28-0.44)
CD8+ Cytotoxic T-cells/µL	0.64 (0.21-1.05)	0.24 (0.13-0.52)	0.36 (0.11-0.42)
B Lymphocytes /µL	0.015 (0-0.04)	0.01 (0.01-0.03)	0.049 (0.03-0.14)
**NK Lymphocytes/µL**	**0.095 (0.07-0.15)†**	**0.29 (0.11-0.40)†**	**0.09 (0.08-0.13)**

A total of 12 patients were enrolled in this study. Samples were only obtained from 10 out of 12 patients at 6 months after treatment and from 8 out of 12 patients at 12 months. Statistical analysis was performed using the Friedman test. Were observed no differences at pre-treatment and 6 months with ruxolitinib († p=0.26). The bold values emphasize the NK cell population, which is the subject of this article.

## Discussion

In this study, we characterized the effects of ruxolitinib on the phenotype, proliferation, and functionality of NK cells activated by the TLR pathway. NK cells express TLRs on their cell surface and can recognize LPS (TLR4 agonist) or aCpG (TLR9 agonist), among others, which release pro-inflammatory cytokines and activate the immune response (23, 35).

As others have shown, we observed that TLR4/9 ligand-activated NK cells increased the expression of the pro-inflammatory cytokines IL-6, IFNγ, and TNFα (36, 37), and ruxolitinib did not completely impair these cytokines’ expression or production. The production of IL-1β by TLR activating NK cells is controversial. Nevertheless, some authors have shown the capacity of activated NK cells to produce IL-1β upon lipoprotein stimulation (38). Also, although production of IL-1β is associated to cells from the innate immune system, cells from the adaptive immune system have shown to release IL-1β after activation through a transcriptional mechanism different from myeloid cells (39). Monocytes, dendritic cells and B cells also express TLR 4 and 9 in their cell surface (40, 41). Myeloid components are commonly involved in cytokine release upon TLR ligand stimulation. In our study, NK cells are the main immune population after NK cell enrichment (**Figure S1**), with expression of some immune cell subsets at very low percentage, like monocytes with a mean of 0.16% (**Figure S1**). We presume most of the response that we observed come from TLR activated NK cells but we cannot rule out that a minority part of the response could come from the activation of other immune cells.

The cytotoxic and degranulation capacities were not abrogated in TLR4/9 ligand-activated NK cells in the presence of ruxolitinib. NK cells’ cytotoxic activity is primarily regulated through JAK1/2, which activates members of the STAT family. These results contrast with those of other authors who observed an inhibition of NK cell cytotoxicity at concentrations of ruxolitinib as low as 0.1 µM when inhibiting cytokine-induced memory-like NK-cell activation directly by the JAK/STAT pathway (11). The increase in the cytotoxic and degranulation capacities of NK cells stimulated with LPS/aCpG was not very high. The level of TLR expression in NK cells is still a matter of debate and can differ in various NK-cell subpopulations (17), with a number of authors stating that TLR4 and TLR9 expression in NK cells is low (35), which could explain this effect. TLR4/9 ligands (LPS and aCpG) did not improve proliferation in NK cells in combination with IL-2. Ruxolitinib decreased the proliferative capacity of NK cells activated with TLR4/9 ligands and IL-2. Other activation mechanisms showed a maintenance of proliferative capacity in the presence of ruxolitinib (11).

Ruxolitinib is not a specific inhibitor of the TLR pathway; cytokine production *via* TLR activation would require higher concentrations of ruxolitinib to inhibit the JAK pathway. As we expected, the functional capacity changes related to ruxolitinib were not due to MyD88 downregulation. MyD88 is an adaptor protein that mediates intracellular signaling of TLRs activated by LPS/aCpG (17). All TLRs signal through the MyD88 adaptor-dependent pathway (except for TLR3) to regulate the expression of cytokines (42). Our data showed that STAT3 was activated in untreated NK cells, as shown in other studies (43, 44). This activation increased after TLR4/9 ligand exposure. Ruxolitinib did not completely inhibit STAT3 phosphorylation in NK cells activated by TLR4 and TLR9; hence, the partial reduction of NK-cell functionality could be explained by pSTAT3 expression at low ruxolitinib concentrations. Also, certain cytokines, such as IL-6, can activate the STAT pathway through the TYK2 receptor (45), which could explain the high IL-6 concentration even when the NK cells were treated with the highest concentration of ruxolitinib. Other JAK independent-signaling pathways can also be involved, as has been reported for the IL-1 family (46) and other cytokines (15, 47). Certain cytokines can also signal through JAK1/3, although this explanation is less likely due to the predominance of the JAK1 protein over JAK3 (48). In contrast, the cytokines dependent on the TLR pathway did not activate STAT5 in NK cells, as shown by the lack of phosphorylation. As expected, IL-2 could activate STAT5 and was inhibited by ruxolitinib (11).

Our data also showed changes in the receptor repertoire on TLR4/9 ligand-activated NK cells. CD69 and CD25 are activating receptors that trigger NK-cell cytotoxicity (49, 50). As has been reported, we also observed that activation *via* TLRs increased the expression of the activation markers CD25 and CD69 (17, 22). As expected, NKG2D was not activated by TLR ligands (43). NKp44 expression increases *via* TLR2-mediated interaction of NK cells (18). Our data showed evidence that TLR4 and TLR9 are not involved in this pathway. Also, in accordance with previous publications, we observed that high ruxolitinib concentrations decreased NKG2D expression through STAT3 inhibition (44). CXCR4 expression in NK cells is involved in NK-cell migration to bone marrow. A number of authors have shown that high CXCR4 expression levels in infused NK cells was associated with an increased probability of objective response in patients with relapsed/refractory acute myeloid leukemia or high-risk myelodysplastic syndrome (51). According to our data, CXCR4 was not affected by TLR ligands as previously reported (11), and, more importantly, ruxolitinib did not inhibit CXCR4 expression in NK cells. Ruxolitinib decreases activating and inhibitory receptors in NK cells activated by TLR ligands (LPS/aCpG). Our data showed that the CD56^+^ CD16^+^ population decreased after TLR stimulation, but the presence of ruxolitinib modified the expression of the CD16 receptor. TLR4/9 ligand-stimulated NK cells showed increased expression of the CD56^bright^ subpopulation. This change could also be involved in increased cytokine production after activation with TLR ligands (52).

**Figure 8:**
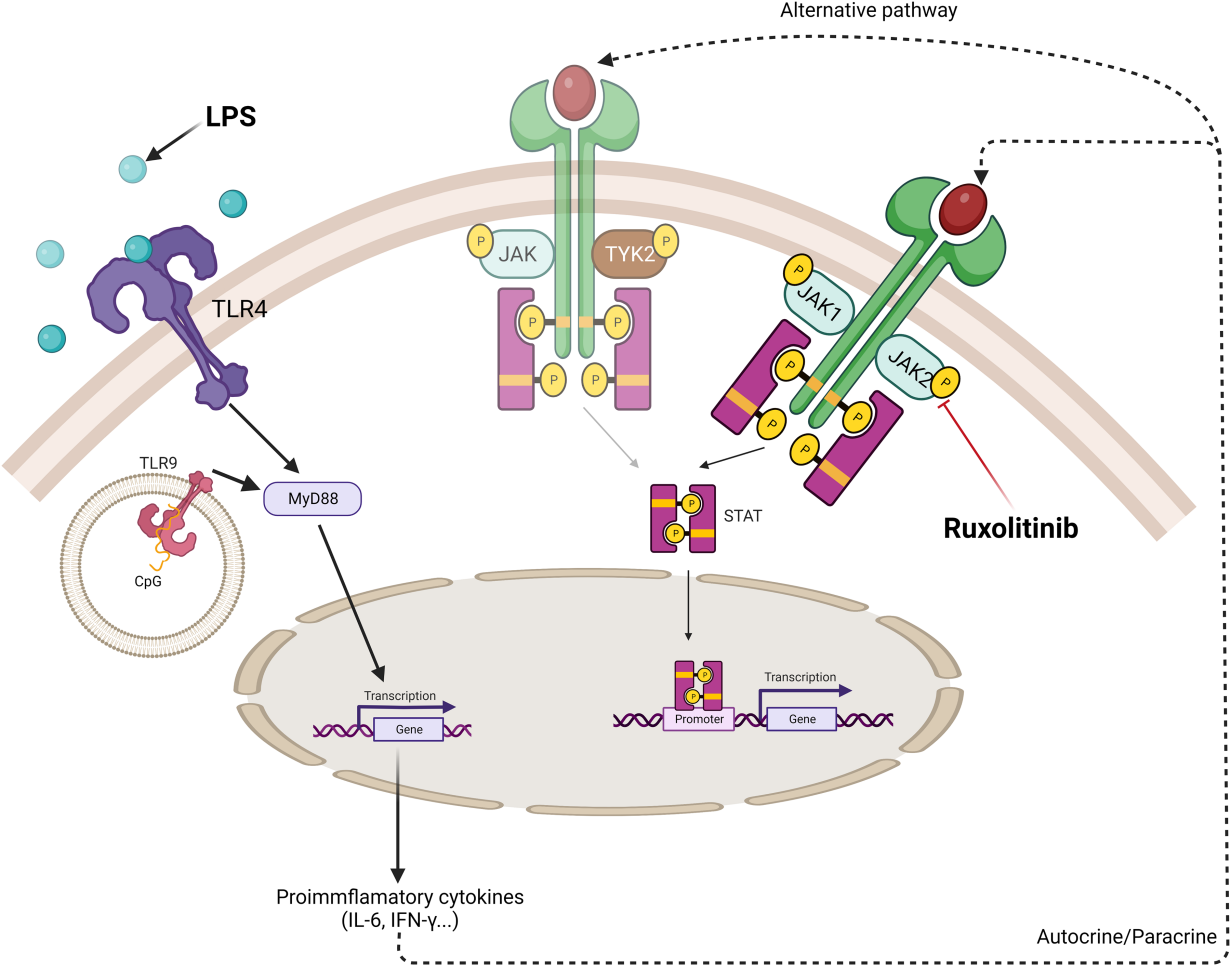
Schematic model of cytokine production in TLR4/9 ligand-activated NK cells and the effect of ruxolitinib in the inhibition of activation of JAK/STAT pathway. TLR receptors present on NK cells recognize specific ligands, bacterial lipopolysaccharide (LPS) and oligodeoxyribonucleotide containing non-methylated CpG motifs (aCpG ODNs). The ligand-receptor complex allows through the adaptor protein, MyD88, the downstream signaling pathway to produce proinflammatory cytokines. Autocrine or paracrine secretion allow these cytokines to be recognized by their receptor on the surface of NK cells activating the JAK/STAT specific pathway. STAT3 phosphorylation is observed when NK cells are activated via TLR ligands. Ruxolitinib, a specific JAK1/JAK2 inhibitor, decreases STAT3 phosphorylation. Other alternative JAK independent-signaling pathways can be involved in the STAT3 phosphorylation by these cytokines such as TYK2. Created with BioRender.com.

We propose that the pro-inflammatory cytokines produced by TLR ligand (LPS/aCpG) stimulation bind to their ligands on NK cells. This binding would activate the JAK/STAT pathway, which could be inhibited in the presence of ruxolitinib. Given that we did not observe an inhibition of phosphorylation, there could be alternate pathways that maintain STAT phosphorylation by these cytokines (**Figure 8**).

Ruxolitinib has a positive effect on SR-GVHD in patients after HSCT (27, 53–56). In the context of GvHD, we need strategies that minimize this adverse effect without affecting the beneficial effect provided by GvL. In patients with myeloproliferative neoplasms in which a constitutive activation of JAK2-dependent signaling occurs, ruxolitinib impairs NK cell function (25). Data on the effect of ruxolitinib on NK cell numbers in the pediatric population are scarce; previous clinical reports found no significant changes (27, 57). In our small cohort of patients, we observed no deleterious effect by ruxolitinib on NK cell numbers. We observed that ruxolitinib did not inhibit NK-cell expansion during short treatment times, whereas T-cell subpopulations tended to decrease. We hypothesize that patients receiving ruxolitinib maintain the GvL effect, either due to the presence of GvHD or due to the role of NK cells in mediating GvL (58).

In this study, our data provide evidence that ruxolitinib has a mild effect on NK-cell activation and functionality by LPS/aCpG cytokine production. These data could have clinical implications for the protective role of NK cells in the GvL effect.

### Limitations

Regarding the limitations of our work, we have not studied the expression of TLRs or mean fluoresce intensity on NK cells. Also, STAT1 is the main member of the STAT family activated by IFNγ. Although we have not analyzed the changes in STAT1 phosphorylation, we would expect to also observe a decrease in STAT1 phosphorylation after ruxolitinib treatment (59).

## Data availability statement

The original contributions presented in the study are included in the article/**Supplementary Material**. Further inquiries can be directed to the corresponding author.

## Ethics statement

The studies involving human participants were reviewed and approved by Ethics Committee of University Hospital La Paz. Written informed consent to participate in this study was provided by the participants’ legal guardian/next of kin.

## Author contributions

AP-M proposed and conceived the original idea. CM-D performed the laboratory experiments. CM-C and LP were in charge of blood collection and processing. AP-M and CF contributed to the interpretation of the results. CM-D and CF wrote the manuscript. CF and AP-M supervised the study. All authors revised the manuscript, participated in the interpretation of the data, and the approval and submission of the manuscript.

## Supplementary material

Supplementary Figure 1 Immune cells ratio before and after use of RosetteSep™ Human NK Cell Enrichment Cocktail in buffy coat from healthy donors. The immune populations were defined as: NK cells (CD45^+^ CD3^-^ CD56^+^), T cells (CD45^+^ CD3^+^ CD56^-^), NKT cells (CD45^+^ CD3^+^ CD56^+^), B cells (CD45^+^ CD19^+^) and monocytes (CD45^+^ CD14^+^). N=8 from 8 different healthy donors.

Supplementary Figure 2 Representative dot plots of gating strategy for the determination of NK cells purity using the RosetteSep™ Human NK Cell Enrichment Cocktail in buffy coat from healthy donors. **(A)** Different percentages of the immune populations (NK cells, T cells, NKT cells, B cells and monocytes) present in the buffy coat prior to enrichment. **(B)** Different percentages of the immune populations (NK cells, T cells, NKT cells, B cells and monocytes) present in the NK cells enrichment. The immune populations were defined as: NK cells (CD45^+^ CD3^-^ CD56^+^), T cells (CD45^+^ CD3^+^ CD56^-^), NKT cells (CD45^+^ CD3^+^ CD56^+^), B cells (CD45^+^ CD19^+^) and monocytes (CD45^+^ CD14^+^). The squares represent the positive population for each receptor analyzed. The percentages of expression of each population are shown.

Supplementary Figure 3 Representative dot plots of CD25, CD69, NKG2D, NKp46 and NKp44 expression by flow cytometry on TLR4/9 ligands-activated NK cells in the absence of ruxolitinib or the presence of increasing concentrations of ruxolitinib. The percentages of expression of each receptor are shown.

Supplementary Figure 4 Representative dot plots of TIGIT, LAG-3, TIM-3, TACTILE and CXCR4 expression by flow cytometry on TLR4/9 ligands-activated NK cells in the absence of ruxolitinib or the presence of increasing concentrations of ruxolitinib. The percentages of expression of each receptor are shown.

Supplementary Figure 5 Representative example of gating strategy of total NK cells (CD56^+^ CD16^+^) and NK cell subsets (CD56^dim^, CD56^bright^, CD56^dim^ CD16^+^ and CD56^bright^ CD16^-^) shown on figure 7. NK cells were activated for 16 hours with 100 ng/mL LPS and 10 µg/ml aCpG in the presence or absence of indicated concentrations of ruxolitinib after overnight incubation NK cells were acquired by flow cytometry.

Supplementary Figure 6 Fold change of NK cells levels from time of HSCT at different post-transplant time points. Patients were divided into two groups, a cohort of 5 patients who received ruxolitinib early post-transplant (within 6 months post-HSCT) **(A)** and a cohort of 4 patients who received ruxolitinib later post-transplant (≥ 6-months post-HSCT) **(B)**. **(A)** Treatment with ruxolitinib early post-HSCT resulted in a reduction of NK cells after treatment in two of the 5 patients analyzed (patients#5 and #7) while in the three remaining patients (patients#3, #4 and #6) NK cells increased after ruxolitinib treatment. **(B)** In the other group, three of the 4 patients who received ruxolitinib showed a reduction in NK cells after treatment (patients#1, #8, and #10) whereas in one patient (patient#2) NK cells increased greatly after treatment.
