# Supplementary figures and images for: Ruxolitinib does not completely abrogate the functional capabilities of TLR4/9 ligand-activated NK cells
Source: Front Immunol. 2023 Jan 5;13:1045316. doi: 10.3389/fimmu.2022.1045316 (PMC9851469; doi:10.3389/fimmu.2022.1045316)

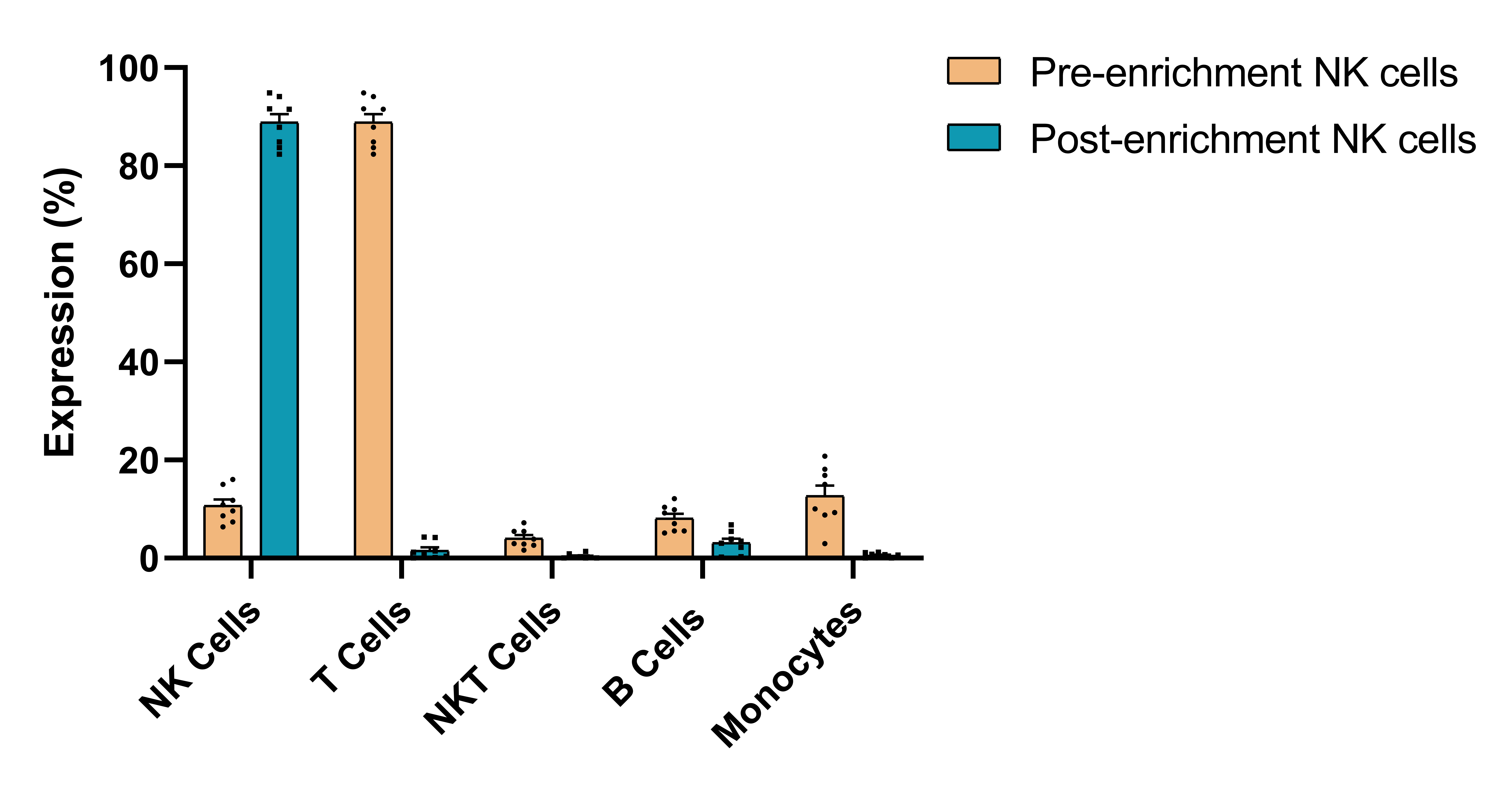

Supplement: Supplementary Figure 1 — Immune cells ratio before and after use of RosetteSep™ Human NK Cell Enrichment Cocktail in buffy coat from healthy donors. The immune populations were defined as: NK cells (CD45+ CD3- CD56+), T cells (CD45+ CD3+ CD56-), NKT cells (CD45+ CD3+ CD56+), B cells (CD45+ CD19+) and monocytes (CD45+ CD14+). N=8 from 8 different healthy donors. [file Image_1.tif]

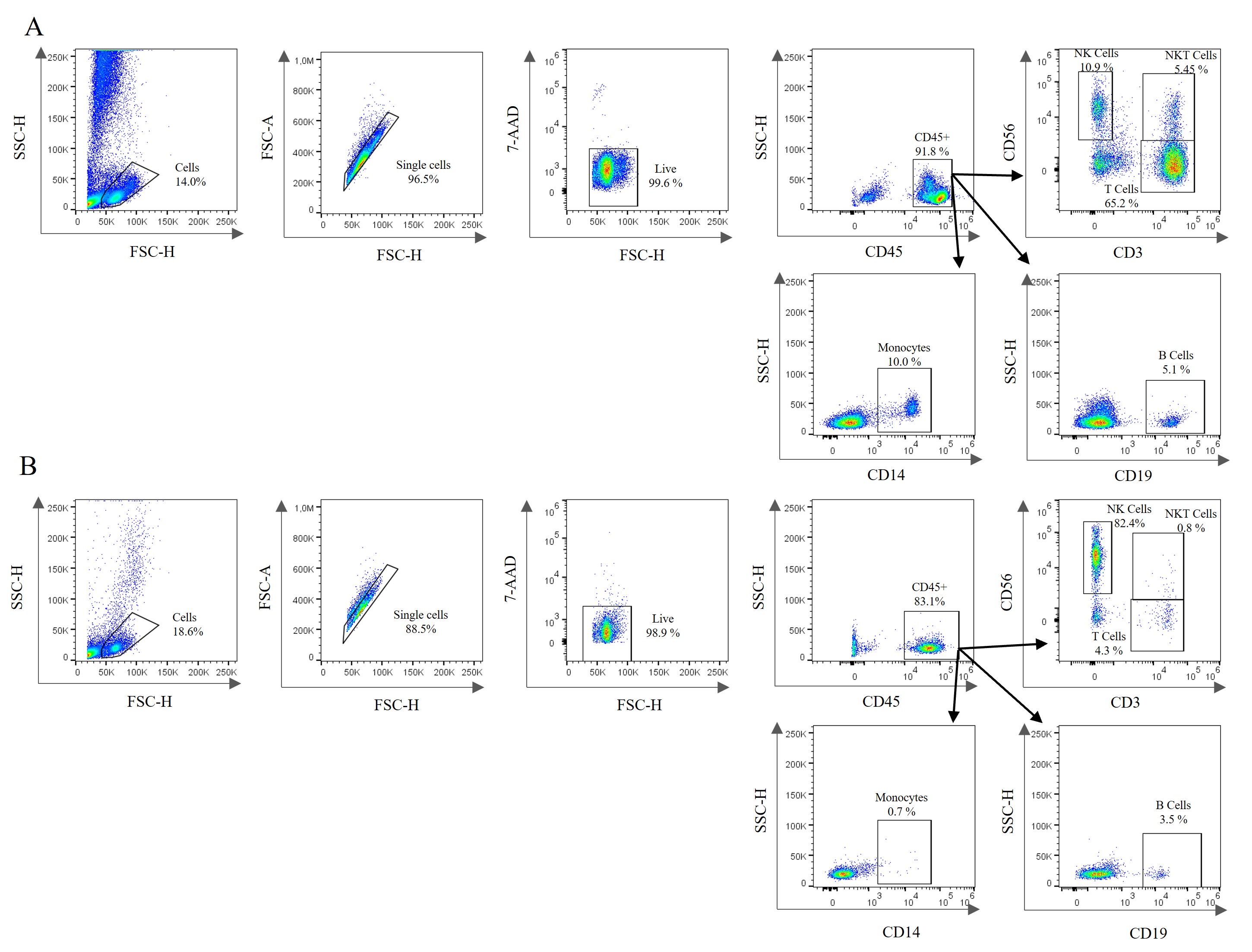

Supplement: Supplementary Figure 2 — Representative dot plots of gating strategy for the determination of NK cells purity using the RosetteSep™ Human NK Cell Enrichment Cocktail in buffy coat from healthy donors. (A) Different percentages of the immune populations (NK cells, T cells, NKT cells, B cells and monocytes) present in the buffy coat prior to enrichment. (B) Different percentages of the immune populations (NK cells, T cells, NKT cells, B cells and monocytes) present in the NK cells enrichment. The immune populations were defined as: NK cells (CD45+ CD3- CD56+), T cells (CD45+ CD3+ CD56-), NKT cells (CD45+ CD3+ CD56+), B cells (CD45+ CD19+) and monocytes (CD45+ CD14+). The squares represent the positive population for each receptor analyzed. The percentages of expression of each population are shown. [file Image_2.jpeg]

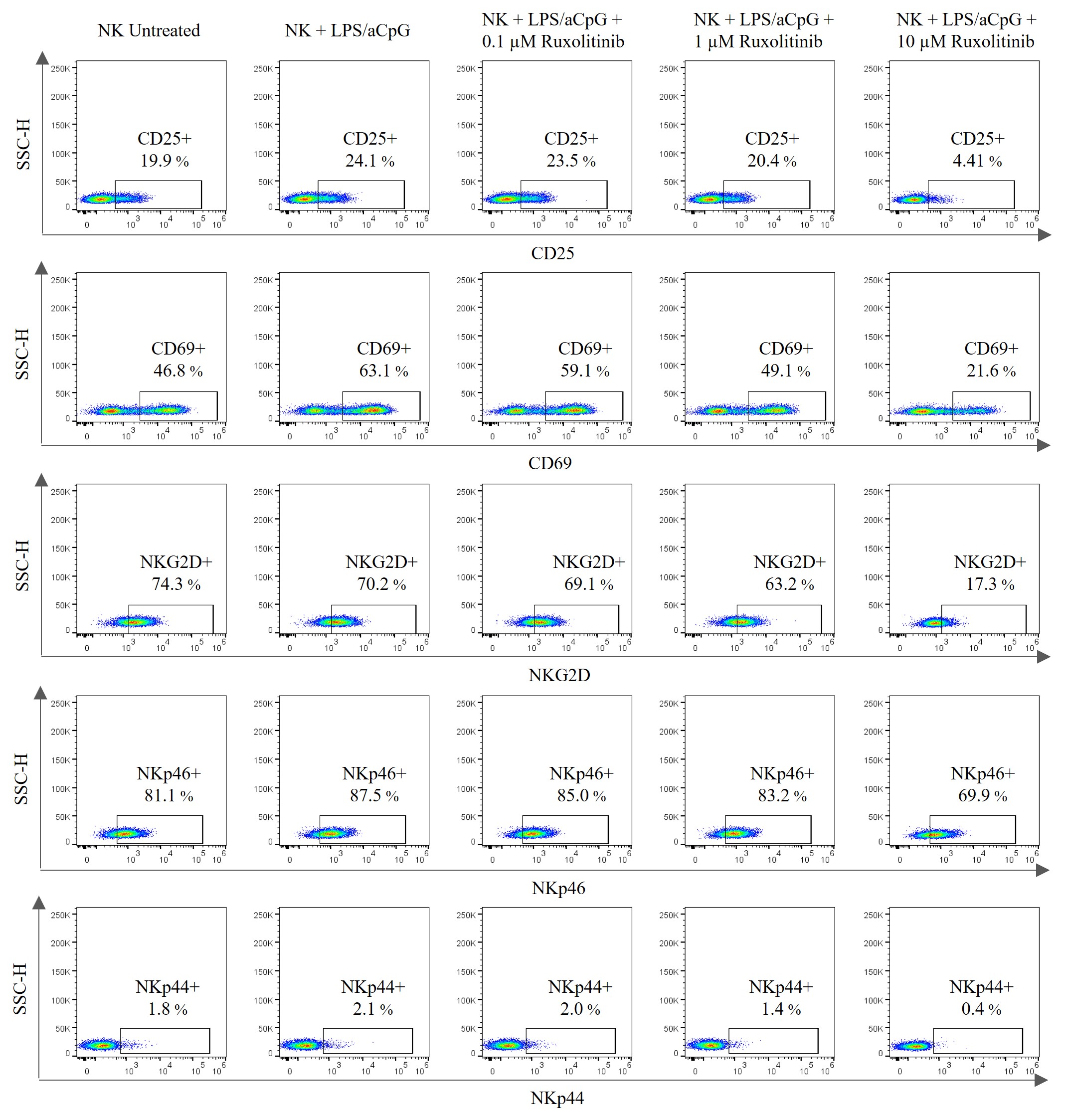

Supplement: Supplementary Figure 3 — Representative dot plots of CD25, CD69, NKG2D, NKp46 and NKp44 expression by flow cytometry on TLR4/9 ligands-activated NK cells in the absence of ruxolitinib or the presence of increasing concentrations of ruxolitinib. The percentages of expression of each receptor are shown. [file Image_3.jpeg]

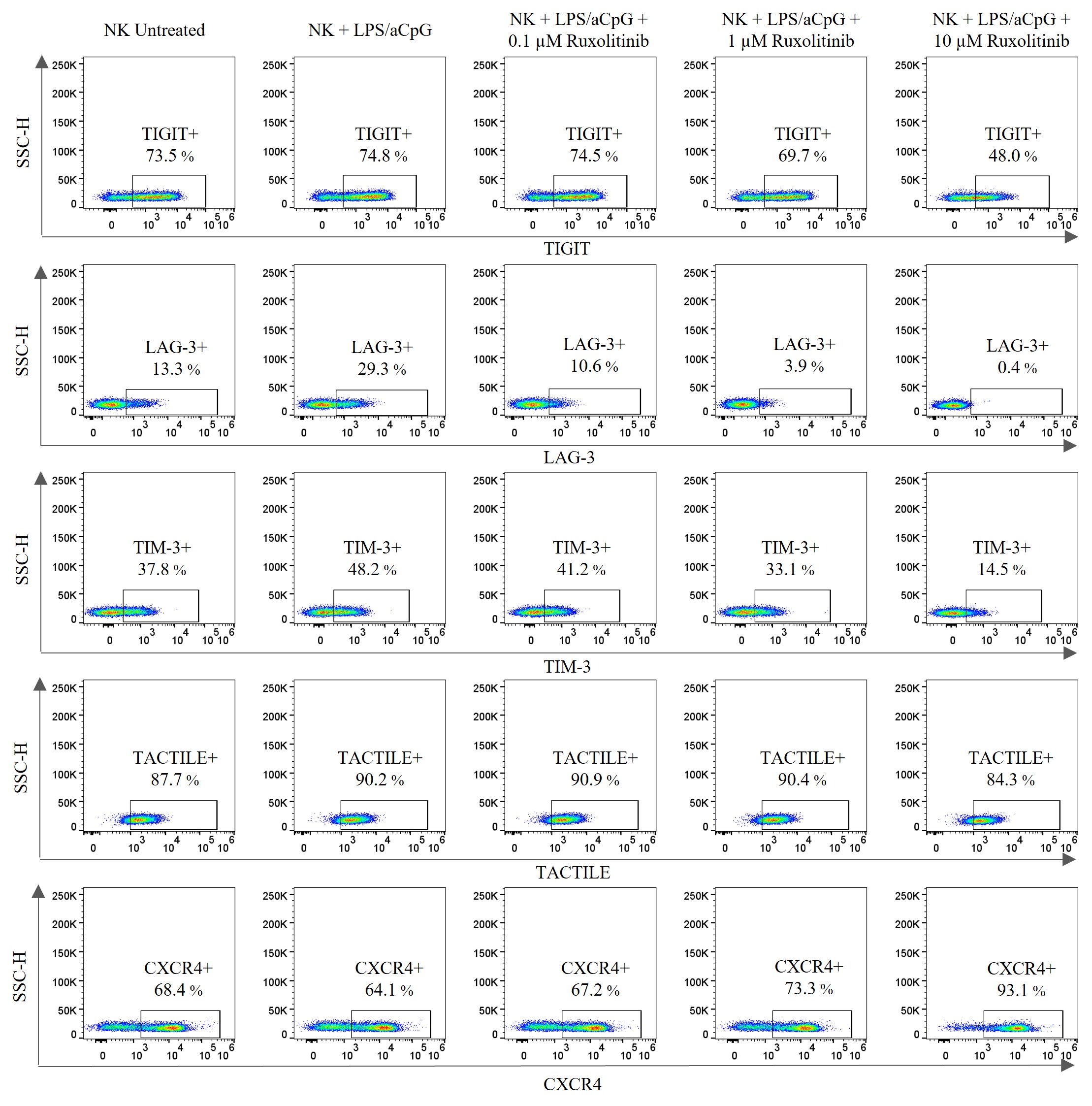

Supplement: Supplementary Figure 4 — Representative dot plots of TIGIT, LAG-3, TIM-3, TACTILE and CXCR4 expression by flow cytometry on TLR4/9 ligands-activated NK cells in the absence of ruxolitinib or the presence of increasing concentrations of ruxolitinib. The percentages of expression of each receptor are shown. [file Image_4.jpeg]

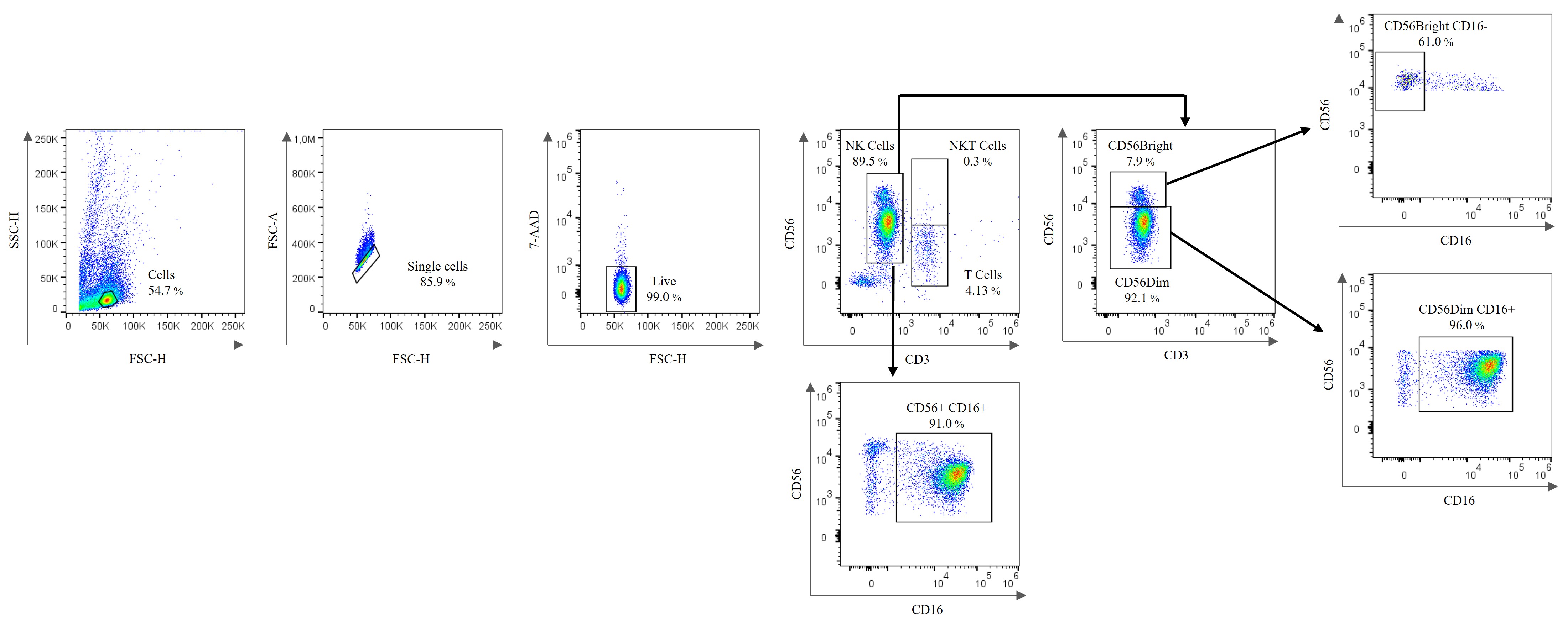

Supplement: Supplementary Figure 5 — Representative example of gating strategy of total NK cells (CD56+ CD16+) and NK cell subsets (CD56dim, CD56bright, CD56dim CD16+ and CD56bright CD16-) shown on figure 7. NK cells were activated for 16 hours with 100 ng/mL LPS and 10 µg/ml aCpG in the presence or absence of indicated concentrations of ruxolitinib after overnight incubation NK cells were acquired by flow cytometry. [file Image_5.jpeg]

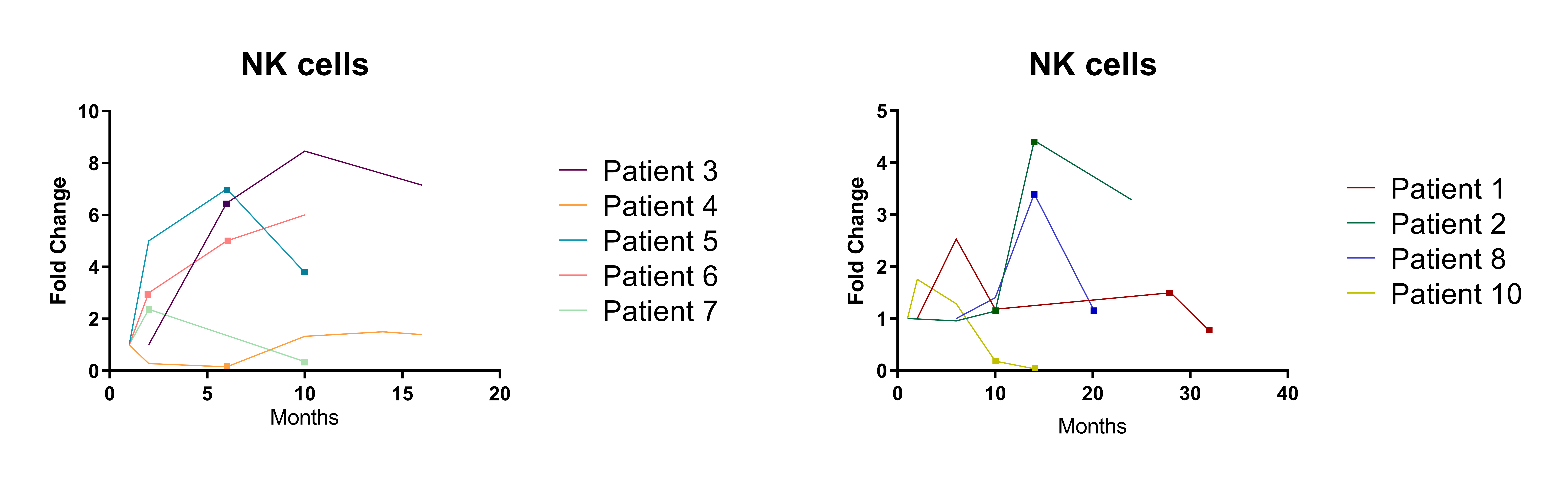

Supplement: Supplementary Figure 6 — Fold change of NK cells levels from time of HSCT at different post-transplant time points. Patients were divided into two groups, a cohort of 5 patients who received ruxolitinib early post-transplant (within 6 months post-HSCT) (A) and a cohort of 4 patients who received ruxolitinib later post-transplant (≥ 6-months post-HSCT) (B). (A) Treatment with ruxolitinib early post-HSCT resulted in a reduction of NK cells after treatment in two of the 5 patients analyzed (patients#5 and #7) while in the three remaining patients (patients#3, #4 and #6) NK cells increased after ruxolitinib treatment. (B) In the other group, three of the 4 patients who received ruxolitinib showed a reduction in NK cells after treatment (patients#1, #8, and #10) whereas in one patient (patient#2) NK cells increased greatly after treatment. [file Image_6.tif]
